# ﻿Morphology and phylogeny reveal two new species and host records of hyphomycetous fungi on *Areca* species from marine habitats in Thailand

**DOI:** 10.3897/mycokeys.118.147229

**Published:** 2025-06-04

**Authors:** Raheleh Asghari, Chayanard Phukhamsakda, E. B. Gareth Jones, Ali Bahkali, Carlo Chris S. Apurillo, Omid Karimi, Pattana Kakumyan, Kevin D. Hyde

**Affiliations:** 1 School of Science, Mae Fah Luang University, Chiang Rai 57100, Thailand; 2 Center of Excellence in Fungal Research, Mae Fah Luang University, Chiang Rai 57100, Thailand; 3 Botany and Microbiology Department, College of Science, King Saud University, Riyadh, 1145, Saudi Arabia; 4 Department of Science and Technology–Center for Research in Science and Technlology (CReST), Philippine Science High School-Eastern Visayas Campus, Palo, Leyte 6501, Philippines; 5 Microbial Products and Innovations Research Group, Mae Fah Luang University, Chiang Rai 57100, Thailand

**Keywords:** 2 new species, marine fungi, molecular phylogeny, palm fungi, rocky shore, saprobe

## Abstract

The marine ecosystem is the largest on Earth, supporting a wide variety of organisms. Fungi in this environment are diverse and play promising ecological roles. This study investigated fungi on submerged, decaying plant materials of *Areca* species trapped between rocks in seawater in Prachuap Khiri Khan Province, Thailand. Morphological and multi-gene phylogenetic analysis (LSU-ITS-*tub*2-SSU for *Tetraploa*, ITS-LSU-*tub*2-*rpb*2 for *Rosellinia*, LSU-ITS-*tef*1-α-*rpb*2 for *Musicillium* and LSU-ITS-*act* for *Sarocladium*) revealed two new species: *Tetraploamaritima*, characterized by the presence of a hilum and elongating appendages, and *Roselliniamaritima*, distinguished by a brown to black conidial-like structure composed of interwoven, irregular cells without distinct conidiophores produced in sterile conditions, unlike other *Rosellinia* asexual morphs. Additionally, two new host records (*Musicilliumtheobromae* and *Sarocladiumgamsii*) are documented, with detailed descriptions of their *in vivo* and *in vitro* morphologies. Detailed morphological descriptions and illustrations are provided. This study contributes to the understanding of fungal diversity in marine environments.

## ﻿Introduction

Fungi live in a variety of ecosystems, from terrestrial to aquatic habitats on various substrates such as plant materials (Hyde et al. 2019, 2020, 2024; Bhunjun et al. 2022). They have a high diversity in marine environments and are known for their important roles as well as for their potential use in medicine, bioremediation, biotechnology and industry (Hyde et al. 1998; Bonugli-Santos et al. 2015; Pang et al. 2016; Jones et al. 2019). Despite their importance, marine fungi have not received much attention as compared to terrestrial fungi (Kohlmeyer and Kohlmeyer 1979; Hyde et al. 2000; Jones et al. 2006, 2015, 2019; Abdel-Wahab et al. 2020; Dayarathne et al. 2020). Currently, more than 2000 marine fungal species have been discovered belonging to 11 different phyla (www.marinefungi.org, accessed in April 2025), with Ascomycota as the most recorded phylum (Jones et al. 2019; Calabon et al. 2023). They are mostly reported in their sexual stages, followed by lesser reports of coelomycetous and hyphomycetous asexual morphs (Hyde et al. 2000; Chatmala et al. 2004; Jones et al. 2009).

Several studies have been conducted on saprobic, endophytic and pathogenic fungi on palms (Arecaceae), showing the high diversity of fungi on this host family worldwide (Hyde 1988; Taylor et al. 1999; Taylor and Hyde 2003; Liu et al. 2010; Marin-Felix et al. 2019; Chen et al. 2020; Konta et al. 2020, 2021, 2023; Karimi et al. 2024; Zhang et al. 2024). Arecaceae is an ancient plant family dating back 66–100 million years (Phukhamsakda et al. 2022). It has a global distribution, predominantly in tropical and subtropical regions, across diverse terrestrial and aquatic habitats (Pereira and Phillips 2023). The palm family comprises around 2600 species within 181 genera (Baker and Dransfield 2016). *Areca* is the type genus of Arecaceae, described by Linnaeus (1753), and mostly distributed in tropical islands (www.palmweb.org). *Areca* species thrive in humid tropical environments and are utilized for various purposes, including ornamental and cultural uses (Dransfield et al. 2008; Heatubun et al. 2012).

Fungal association with *Areca* has been studied predominantly with the focus of pathogenic fungi on the economically important species *A.catechu* (betel nut palm) (To-Anun et al. 2009; Phengsintham et al. 2013; Balanagouda et al. 2021; Harahap 2022). Asghari et al. (2023) introduced a new fungal species found on decaying wood of *Areca* species submerged in marine water. In this study, we proceed with the investigation of hyphomycetous saprobic marine fungi on decaying *Areca* in marine habitats in Thailand. Two new species and two new host records are introduced, supported by morphology and molecular data.

## ﻿Materials and methods

### ﻿Sampling and morphological studies

Decaying submerged woody specimens were collected from the rocky shore at Pranburi, Prachuap Khiri Khan Province (Thailand) in October 2022. The specimens were placed in moist plastic bags and incubated in sterile seawater moist chambers after transferring to the laboratory. The moist chambers were treated with sterilized natural seawater regularly. The samples were examined for fungal presence after returning to the laboratory and up to one month of incubation. The fungal macro-characters were examined using a stereomicroscope (Olympus SZX16, Japan) equipped with an Olympus SC180 digital camera (Olympus, Japan). The fungal microscopic features, such as conidiophores, conidiogenous cells and conidia, were examined by preparing microscopic slides using distilled water or lactoglycerol, followed by using a Nikon ECLIPSE Ni compound microscope (Nikon, Japan) equipped with a Nikon DS-Ri2 digital camera (Nikon, Japan). The Tarosoft (R) Image Framework program (Tarosoft, Thailand) was used to measure the fungal characters, and Adobe Photoshop CS6 Extended version 13.1.2 software (Adobe Systems Inc., The United States) was used to process the figures. The single spore isolation method was employed to obtain pure cultures (Senanayake et al. 2020) using potato dextrose agar (PDA). Specimens were deposited at the Mae Fah Luang University Herbarium, Chiang Rai, Thailand (MFLU), and the living cultures were deposited at the Mae Fah Luang University Culture Collection (MFLUCC). The new taxa were linked to the Facesoffungi (Jayasiri et al. 2015) and Index Fungorum databases.

### ﻿DNA extraction, PCR amplification and sequencing

Fresh, pure cultures were selected for DNA extraction. Mycelium was scraped using sterilized surgical blades. Genomic DNA was extracted following the manufacturer’s protocol of a Mega Genomic DNA Extraction Kit (Omega Bio-tek Inc., The United States). Polymerase chain reactions (PCR) were conducted to amplify the internal transcribed spacer region rDNA (ITS), the 28S large subunit rDNA (LSU), the 18S small subunit rDNA (SSU), the RNA polymerase II second largest subunit (*rpb*2) gene, the translation elongation factor 1-alpha (*tef*1-α) gene, and the partial β-tubulin II (*tub*2). Primers and the PCR condition were described as in Table 1. The total volume of the PCR mixture (25 µL) contained double distilled water (ddH_2_O) (9.5 µL), 2X GoTaq® green PCR master mix (PROMEGA, Madison, WI, USA) (12.5 µL), DNA template (1 µL), and 1 µL of each primer (20 µM). The PCR products were checked on 1.7% agarose electrophoresis gels stained with 4S Green Stain. The amplified PCR products were sequenced at SolGent Co. (South Korea).

**Table 1.** Primers and PCR conditions used in the study.

Gene Regions	Primers	PCR conditions	References
**ITS**	ITS5/ITS4	95 °C for 5 min, 35 cycles of 94 °C for 45 s, 53 °C for 45 s, and 72 °C for 2 min, 72 °C for 10 min	White et al. (1990)
**LSU**	LR0R/LR5	94 °C for 4 min, 35 cycles of 95 °C for 45 s, 56 °C for 45 s, and 72 °C for 1 min, 72 °C for 10 min	Vilgalys and Hester (1990); Rehner and Samuels (1994)
**SSU**	NS1/NS4	94 °C for 4 min, 35 cycles of 94 °C for 30 s, 55 °C for 50 s, and 72 °C for 1.30 min, 72 °C for 10 min	White et al. (1990)
***rpb*2**	fRPB2-5f/fRPB2-7cR	95 °C for 5 min, 40 cycles of 95 °C for 1 min, 54 °C, 90 s, and 72 °C for 90 s, 72 °C for 10 min	Liu et al. (1999)
***tef*1-α**	EF1-983F/EF1-2218R	95 °C for 5 min, 40 cycles of 95 °C for 1 min, 54 °C, 90 s, and 72 °C for 90 s, 72 °C for 10 min	Rehner (2001)
***tub*2**	T1/T22	94 °C for 4 min, 35 cycles of 94 °C for 30 s, 55 °C for 50 s, and 72 °C for 1.30 min, 72 °C for 10 min	Glass and Donaldson (1995)

### ﻿Sequence alignment and phylogenetic analyses

The generated sequences were assembled using SeqMan software version 7.1.0. The assembled sequences were searched on the Nucleotide BLAST search (https://blast.ncbi.nlm.nih.gov) to identify the closest taxa. The reference sequences of closely related taxa were obtained from previous publications (Tibpromma et al. 2018; Giraldo and Crous 2019; Hyde et al. 2020; Phukhamsakda et al. 2020; Hou et al. 2023; Li et al. 2024; Zhang et al. 2024; Zhao et al. 2024) and GenBank. The sequences were aligned automatically in MAFFT version 7 (Katoh et al. 2019) and edited manually and/or on the NGPhylogeny.fr website (Lemoine et al. 2019) using the trimAl tool. The datasets were concatenated according to previous research on each genus, using Mesquite version 3.10 (Maddison and Maddison 2016). Maximum likelihood (ML) and Bayesian inference analyses were carried out for both individual loci and combined datasets on the CIPRES Science Gateway portal (CIPRES) (Miller et al. 2010). Maximum likelihood analysis was carried out using RAxMLHPC2 on the XSEDE (v. 8.2.10) tool (Stamatakis 2014) under the GTRCAT substitution model and 1000 bootstrap (B) iterations. Bayesian inference was performed using MrBayes 3.2.2 in CIPRES (Ronquist et al. 2012) to evaluate posterior probabilities (BPP) with four simultaneous Markov chains run for 10,000,000 generation. Trees were sampled every 1000^th^ generations, and the consensus tree and posterior probability were determined after removing the first 25% of the sampled trees in burn-in phase (Larget and Simon 1999). Phylograms were visualized using FigTree version 1.4.0 (Rambaut 2015), and they were adjusted using Adobe Photoshop CS6 Extended version 13.1.2 and Incscape 1.3.3.

## ﻿Results

### ﻿Phylogenetic analyses of *Tetraploa*

**Figure 1.**
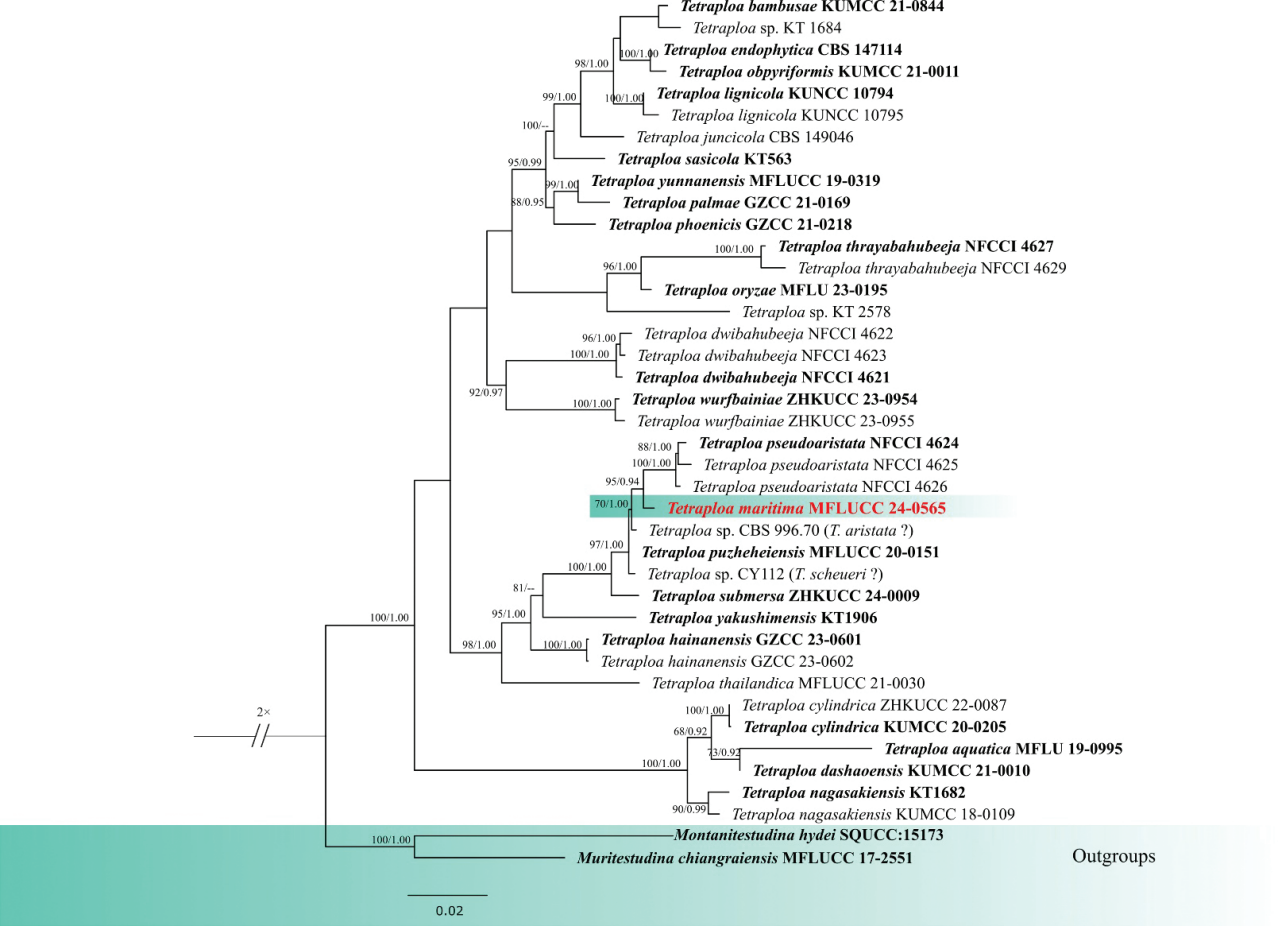
Phylogram generated from maximum likelihood analysis based on combined LSU, ITS, *tub*2 and SSU sequence data of *Tetraploa*. Maximum likelihood bootstrap support ≥ 65% and Bayesian posterior probabilities (BPP) ≥ 0.90 are given near the nodes, respectively. The tree is rooted to *Muritestudinachiangraiensis* (MFLUCC 17-2551) and *Montanitestudinahydei* (SQUCC:15173). The newly generated sequences are indicated in red, and the type strains are indicated in bold.

The combined analysis of LSU, ITS, *tub*2 and SSU sequence data of *Tetraploa* was performed with *Muritestudinachiangraiensis* (MFLUCC 17-2551) and *Montanitestudinahydei* (SQUCC 15173) as the outgroup taxa (Fig. 1). The combined dataset contained 3298 characters (LSU = 1080 bp, ITS = 569 bp, *tub*2 = 638, SSU = 1011) after alignments, including the gaps. Analysis of the combined dataset yielded a best-scoring tree with an optimal log-likelihood value of -12408.104565 (Fig. 1). The alignment had 41 sequences with 812 distinct patterns. Estimated base frequencies were as follows: A = 0.247947, C = 0.249495, G = 0.273749, T = 0.228808, Rate parameters: AC = 3.650751; AG = 3.979381; AT = 2.302782; CG = 1.799753; CT = 9.619686; GT = 1.000000; Gamma shape alpha = 0.122858. After discarding the first 25% of generations in the Bayesian analyses, 7500 trees were remained, from which the 50% consensus tree and posterior probabilities were determined.

### ﻿Phylogenetic analyses of Xylariaceae

**Figure 2.**
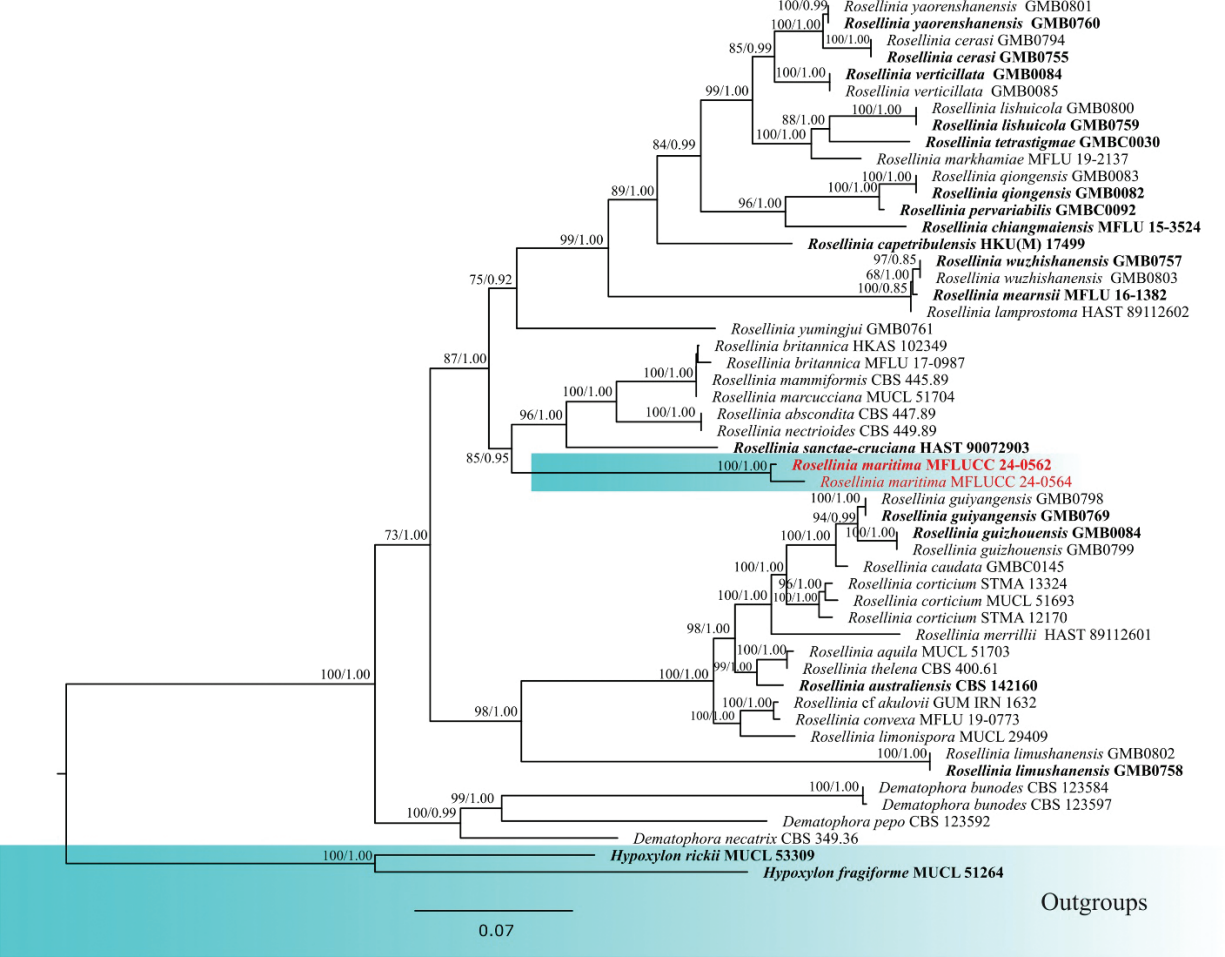
Phylogram generated from maximum likelihood analysis based on combined ITS, LSU, *tub*2 and *rpb*2 sequence data of Xylariaceae. Maximum likelihood bootstrap support ≥ 65% and Bayesian posterior probabilities (BPP) ≥ 0.90 are given near the nodes, respectively. The tree is rooted to *Hypoxylonrickii* (MUCL 53309) and *H.fragiforme* (MUCL 51264). The newly generated sequences are indicated in red, and the type strains are indicated in bold.

The combined analysis of ITS, LSU, *tub*2 and *rpb2* sequence data of Xylariaceae was performed with *Hypoxylonrickii* (MUCL 53309) and *H.fragiforme* (MUCL 51264) as the outgroup taxa (Fig. 2). The combined dataset contained 5441 characters (ITS = 560 bp, LSU = 860 bp, *tub*2 = 2900, *rpb*2 = 1121) after alignments, including the gaps. Analysis of the combined dataset yielded a best-scoring tree with an optimal log-likelihood value of -37958.515421 (Fig. 2). The alignment had 52 sequences with 2206 distinct patterns. Estimated base frequencies were as follows: A = 0.222688, C = 0.282274, G = 0.251124, T = 0.243914, Rate parameters: AC = 1.130042; AG = 4.802871; AT = 1.154978; CG = 0.881707; CT = 6.304393; GT = 1.000000; Gamma shape alpha = 0.238462. After discarding the first 25% of generations in the Bayesian analyses, 7500 trees remained, from which the 50% consensus tree and posterior probabilities were determined.

### ﻿Phylogenetic analyses of Plectosphaerellaceae

**Figure 3.**
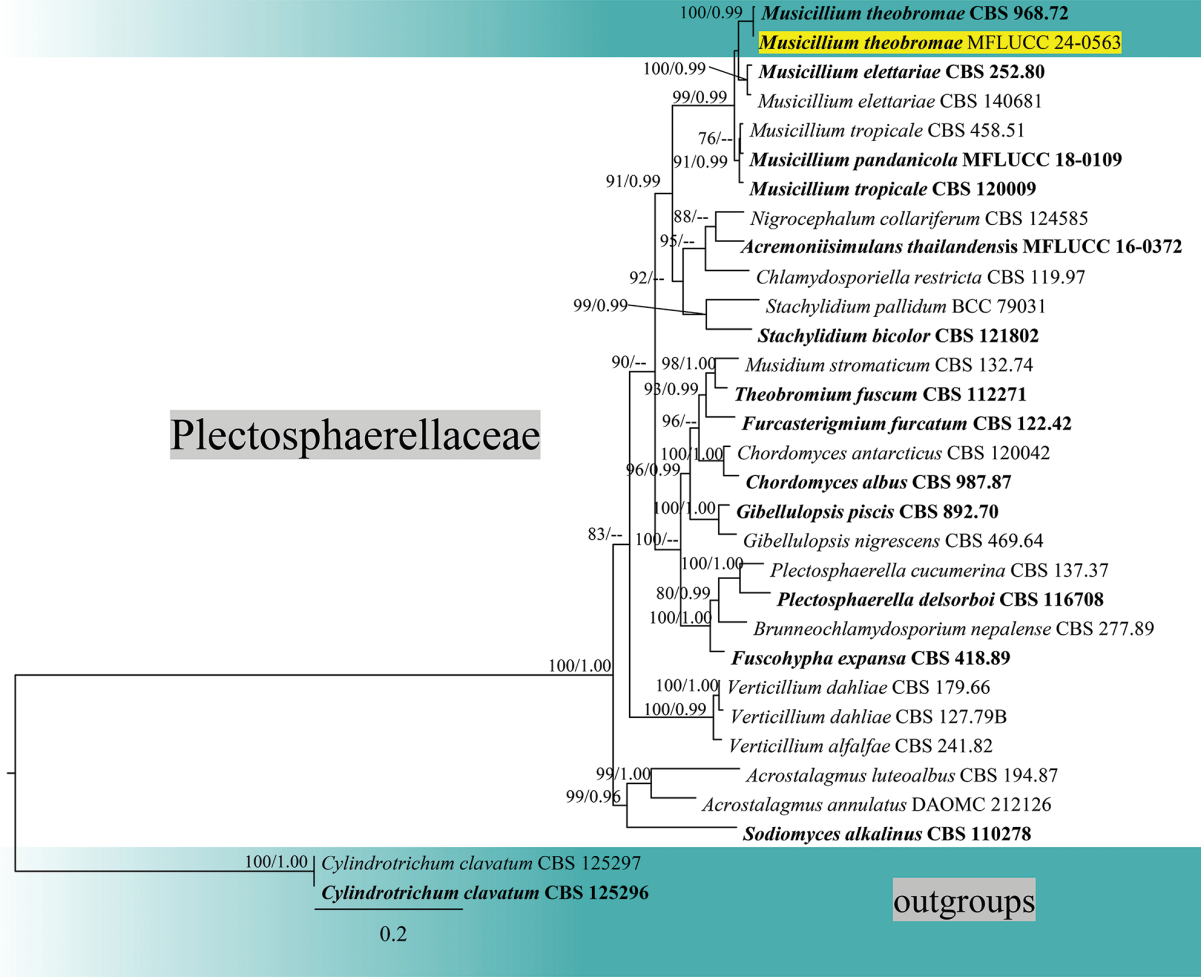
Phylogram generated from maximum likelihood analysis based on combined LSU, ITS, *tef*1-α and *rpb*2 sequence data of Plectosphaerellaceae. Maximum likelihood bootstrap support ≥ 65% and Bayesian posterior probabilities (BPP) ≥ 0.90 are given near the nodes, respectively. The tree is rooted to *Cylindrotrichumclavatum* (CBS 125296, CBS 125297). The newly generated sequence is highlighted in yellow and the type strains are indicated in bold.

**Figure 4.**
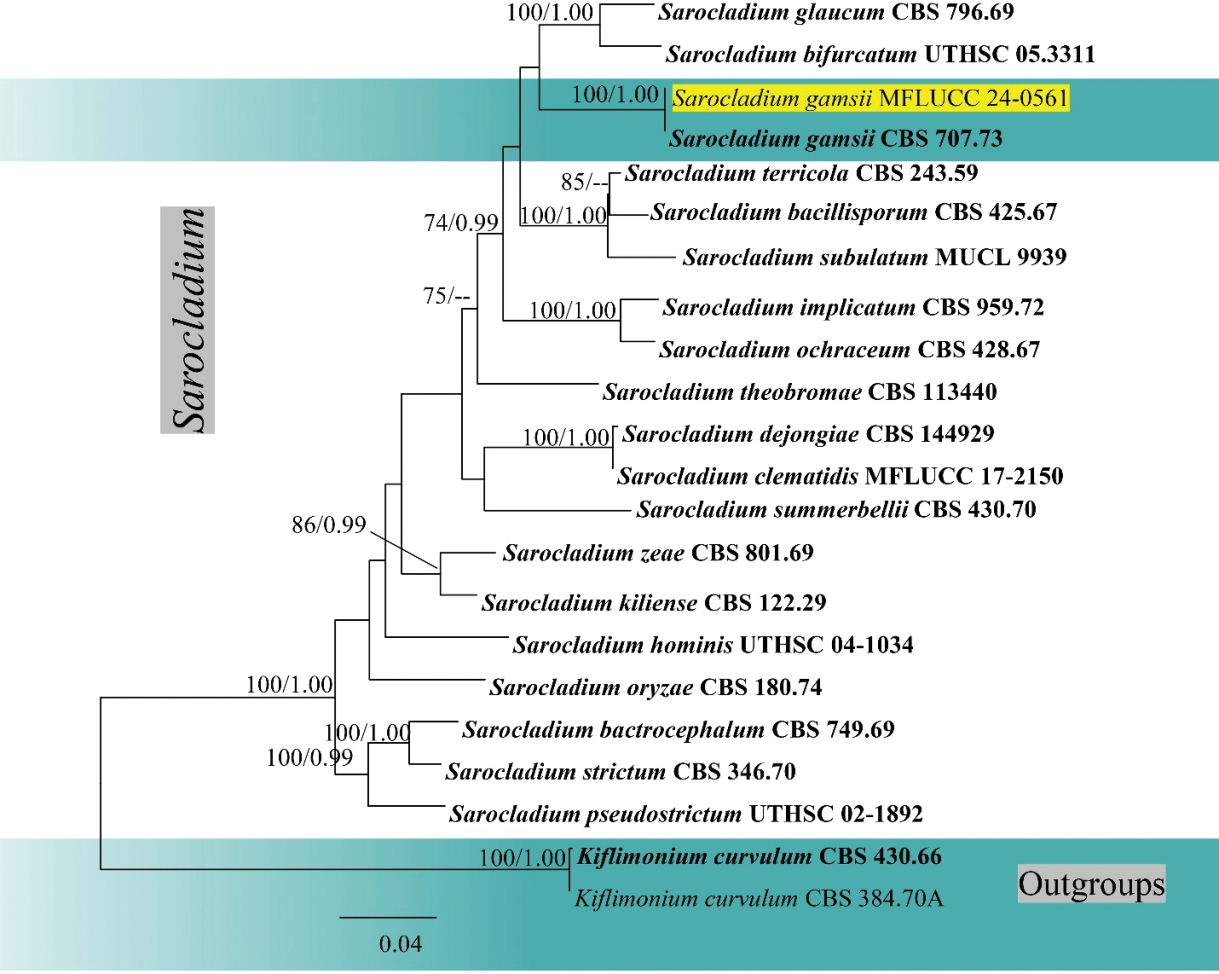
Phylogram generated from maximum likelihood analysis based on combined LSU, ITS and *act* sequence data of *Sarocladium*. Maximum likelihood bootstrap support ≥ 65% and Bayesian posterior probabilities (BPP) ≥ 0.90 are given near the nodes, respectively. The tree is rooted to *Kiflimoniumcurvulum* (CBS 430.66, CBS 384.70A). The newly generated sequence is highlighted in yellow, and the type strains are indicated in bold.

The combined analysis of LSU, ITS, *tef*1-α and *rpb*2 sequence data of Plectosphaerellaceae was performed with *Cylindrotrichumclavatum* (CBS 125296, CBS 125297) as the outgroup taxa (Fig. 3). The combined dataset contained 3,269 characters (LSU = 870 bp, ITS = 544 bp, *tef*1-α = 858, *rpb*2 = 997) after alignments, including the gaps. Analysis of the combined dataset yielded a best-scoring tree with an optimal log-likelihood value of -18111.606810 (Fig. 4). The alignment had 31 sequences with 1017 distinct patterns. Estimated base frequencies were as follows: A = 0.224951, C = 0.295999, G = 0.282023, T = 0.197027, Rate parameters: AC = 0.696864; AG = 1.667863; AT = 1.053040; CG = 0.729378; CT = 4.900764; GT = 1.000000; Gamma shape alpha = 0.194519. After discarding the first 25% of generations in the Bayesian analyses, 7500 trees were remained from which the 50% consensus tree and posterior probabilities were determined.

### ﻿Phylogenetic analyses of *Sarocladium*

The combined analysis of LSU, ITS and *act* sequence data of *Sarocladium* was performed with *Kiflimoniumcurvulum* (CBS 430.66, CBS 384.70A) as the outgroup taxa (Fig. 4). The combined dataset contained 2,319 characters (LSU = 894 bp, ITS = 580 bp, *act* = 845) after alignments, including the gaps. Analysis of the combined dataset yielded a best-scoring tree with an optimal log-likelihood value of -9162.577439 (Fig. 4). The alignment had 22 sequences with 604 distinct patterns. Estimated base frequencies were as follows: A = 0.227911, C = 0.281575, G = 0.275350, T = 0.215164, Rate parameters: AC = 1.495411; AG = 2.342956; AT = 2.214487; CG = 0.793329; CT = 7.315308; GT = 1.000000; Gamma shape alpha = 0.146342. After discarding the first 25% of generations in the Bayesian analyses, 7500 trees were remained, from which the 50% consensus tree and posterior probabilities were determined.

### ﻿Taxonomy

#### ﻿ Tetraploa maritima

Taxon classificationFungiPleosporalesTetraplosphaeriaceae

R. Asghari, Phukhams. & K.D. Hyde sp. nov.

FCB9A7BF-2D85-5EEF-8B9D-5E4E5EDDC19F

Index Fungorum: IF903274

Facesoffungi Number: FoF17211

##### Etymology.

The epithet “*maritima*” refers to the marine habitat where the holotype was collected.

##### Holotype.

MFLU 24-0455.

##### Description.

**Figure 5.**
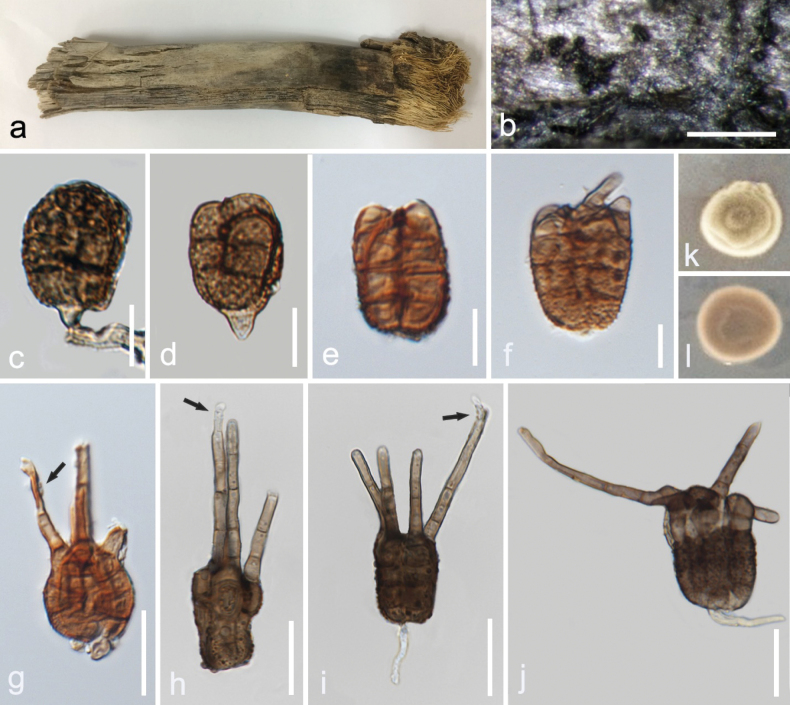
*Tetraploamaritima* (MFLU 24-0455, holotype) **a** colonies on the host substrate **b** close -up of colonies **c** conidiogenous cell and conidium **d–j** conidia (arrows show the ongoing growth of appendages) **k** colony on PDA after 10 days (above) **l** colony on PDA after 10 days (reverse). Scale bares: 100 µm (**b**); 10 µm **(c–f)**; 20 µm **(g–j)**.

***Saprobic*** on decaying branches of *Areca* sp. **Sexual morph**: Undetermined. **Asexual morph**: ***Hyphomycetous*** (*in vivo*, Fig. 5). ***Mycelia*** on natural substrates superficial, branched, septate, sometimes with swollen cells, black or dark brown. ***Conidiophores*** not observed. ***Conidiogenous cells*** 4.5−17 × 1−3 µm (x– = 11 × 2 μm, n = 5) monoblastic, brown, thick walled, integrated. ***Conidia*** 14−28 × 13−18.5 µm (x– = 21.5 × 15 μm, n = 20), solitary, short cylindrical, thick-walled, verrucose to granular, brown, mostly with a subhyaline to pale brown hilum up to 13 µm (–17), with 3–4 columns 5.5−9 µm wide (x– = 7, n = 20), columns are compact and connected almost all their length, 2–4 septa in each column, constricted at septa. ***Appendages*** 19−48 × 2−3 µm (x– = 30 × 2.7 μm, n = 15), 3.5−4.5 µm at the base, setose, unbranched, thick-walled, thin-walled at the apex, up to four septa, finely verrucose, brown, pale brown to subhyaline at the apex, sometimes continuing to grow, forming a subcylindrical narrower paler cell up to 13 µm (n = 10) length.

##### Culture characteristics.

Conidia germinating on PDA within 12 h. Colonies on PDA reaching 1.5 cm diam. after 10 d at 25 ± 2 °C, velvety to floccose, raised, round, entire margin, grayish yellow, with whitish edge, reverse reddish brown with pale buff edge.

##### Material examined.

Thailand, Prachuap Khiri Khan Province, Pranburi, on decaying *Areca* wood submerged in seawater and trapped between rocks, 25 October 2022, K.D. Hyde, R6g (MFLU 24-0455, holotype), ex-type living culture (MFLUCC 24-0565).

##### GenBank numbers.

*Tetraploamaritima*MFLUCC 24-0565 (ex-type): ITS = PQ778934, LSU = PQ778930, SSU = PQ778940, *tub*2 = PQ885482.

##### Notes.

The reconstruction of phylogenies from LSU, ITS, *tub*2 and SSU sequence data showed that *Tetraploamaritima* (MFLUCC 24-0565) formed a distinct clade with *T.pseudoaristata* (NFCCI 4624, NFCCI 4625, NFCCI 4626) in both maximum likelihood and Bayesian analyses with 95% ML/0.94 BPP statistical support. Both maximum likelihood and Bayesian analyses showed the same topology. The closest match of the ITS sequence of *Tetraploamaritima* (MFLUCC 24-0565) was 98.92% similar across 100% of the query sequence to *T.pseudoaristata* (NFCCI 4626). In a BLAST search in GenBank, the closest match of the LSU sequence of *T.maritima* (MFLUCC 24-0565) was 99.88% similar to *T.pseudoaristata* (NFCCI 4624, NFCCI 4625, NFCCI 4626). *Tetraploamaritima* (MFLU 24-0455) has similar morphology to *T.pseudoaristata* (NFCCI 4624) by having verrucose conidia with four apical appendages but differs in having a distinguishable hilum and shorter and narrower appendages (19−48 × 2−3 µm vs. 23–107.5 × 2.4–5.2 µm), which are sometimes elongate above the apex, unlike *T.pseudoaristata* (NFCCI 4624). Conidial columns in *T.maritima* (MFLU 24-0455) are compact and connected almost fully along the length, while in *T.pseudoaristata* (NFCCI 4624) they are partly split, with the two columns totally separating at the upper part (Hyde et al. 2020). *Tetraploamaritima* (MFLUCC 24-0565) showed 2.7% (16 out of 595), 0.2% (2 out of 874) and 2.4% (10 out of 423) base pair differences with *T.pseudoaristata* (NFCCI 4624) in ITS, LSU and *tub*2 respectively, without gaps. Therefore, *T.maritima* (MFLU 24-0455) is introduced as a novel species based on morphological and molecular evidence.

#### ﻿ Rosellinia maritima

Taxon classificationFungiXylarialesXylariaceae

R. Asghari, Phukhams. & K.D. Hyde sp. nov.

A63FEA12-A51F-5F1E-8DE0-C72846229B2C

Index Fungorum: IF903275

Facesoffungi Number: FoF17212

##### Etymology.

The epithet “*maritima*” refers to the marine habitat where the holotype was collected.

##### Holotype.

MFLU 24-0452.

##### Description.

**Figure 6.**
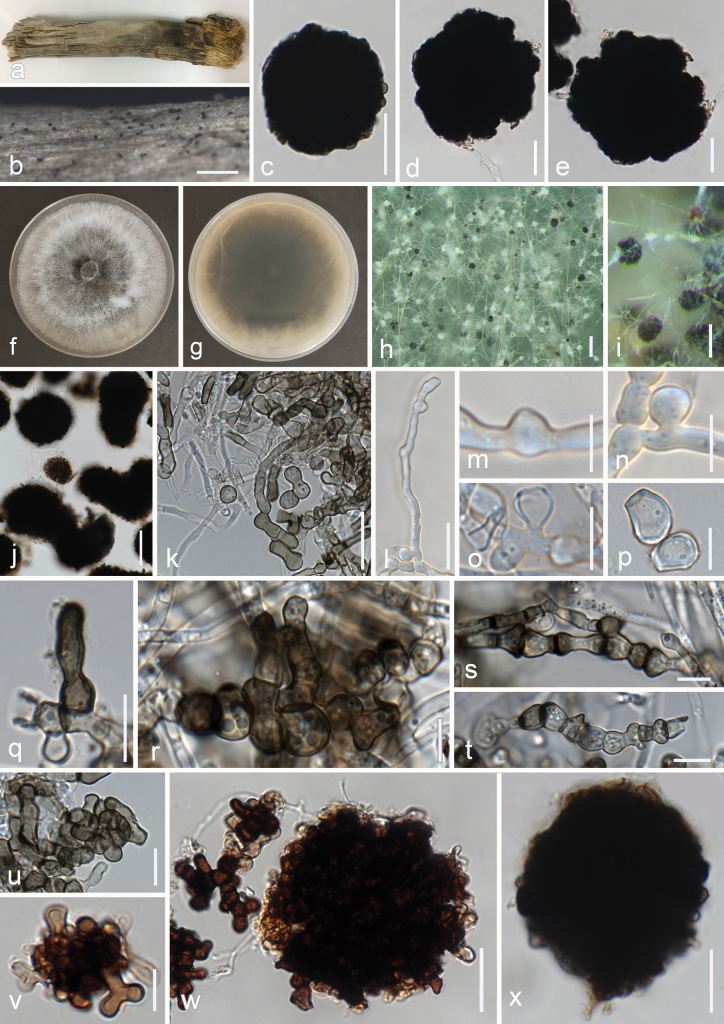
*Roselliniamaritima* (MFLU 24-0452, holotype) **a** colonies on substrate **b** close-up of colonies on substrate **c–e** sterile globules (conidial-like elements) **f–x***Roselliniamaritima* (MFLUCC 24-0562, ex-type) **f, g** colony on PDA (above and reverse) **h, i** close-up of colonies in culture **j** sterile globules in culture **k, l** mycelia **m–o** developing aleurioconidia **p** aleurioconidia **q–t** developing sterile hyphal elements **u–x** developing sterile globules. Scale bars: 500 µm (**b**); 250 (**h**); 100 (**i**); 50 (**j**); 20 µm (**c–e, k–l, w,x**); 10 µm (**m–v**).

***Saprobic*** on decaying branches of *Areca* sp. **Sexual morph**: Undetermined. **Asexual morph**: ***Hyphomycetous*** (*in vivo*, Fig. 6a–e). ***Colonies*** on natural substrates superficial, effuse, scattered, black. ***Mycelia*** on natural substrates superficial to immersed, branched, septate, subhyaline to brown. ***Sterile globules*** 52−74 × 47−69 µm (x– = 62 × 56 μm, n = 20), conidial-like, produced laterally or terminally on the hyphae, solitary, globose to subglobose or irregular, muriform appearance, composed of interwoven, unevenly arranged cells with irregular shapes, thick-walled, dark brown to black, darker in the center. ***Hyphomycetous*** (*in vitro*, Fig. 6f–x). ***Colonies*** on PDA. ***Vegetative hyphae*** subhyaline, pale brown to brown, branched, septate, simple or sometimes irregularly moniliform. ***Conidiophores*** not seen or micronematous. ***Conidiogenous cells*** integrated, up to 13 μm in length, subcylindrical, subhyaline to brown. ***Conidia*** 8−13 × 5−10 µm (x– = 10.5 × 7.5 μm, n = 15), aleurioconidia, solitary, obovoid, truncate base, hyaline to brown, thick-walled, guttulate, intercalary, laterally or terminally, initiating by hyphal inflammation and separating from hyphae by forming septa. ***Sterile globules*** 37−145 × 31−66 µm (x– = 79 × 49 μm, n = 20), conidial-like, produced laterally or terminally on the hyphae, solitary, globose to subglobose, oblong or irregular, muriform appearance, agglomerations of hyphal structures, composed of interwoven, unevenly arranged cells with irregular shapes, thick wall, dark brown to black, darker in the center.

##### Culture characteristics.

Conidia germinating on PDA within 12 h. Colonies on PDA reaching 5 cm diam. after one month at 25 ± 2 °C, felty, flat, round, entire margin, dull, gray and white concentric rings, with dirty whitish edge, reverse dark brown with olivaceous gray edge.

##### Material examined.

Thailand, Prachuap Khiri Khan Province, Pranburi, on decaying *Areca* wood submerged in seawater and trapped between rocks, 25 October 2022, K.D. Hyde, R6c (MFLU 24-0452, holotype), isotype (MFLU 24-0453), ex-type living culture (MFLUCC 24-0562), ex-isotype living culture (MFLUCC 24-0564).

##### GenBank numbers.

*Roselliniamaritima*MFLUCC 24-0562 (ex-type): ITS = PQ778938, LSU = PQ778933, *tub*2 = PQ885479, *rpb*2 = PQ885483; *Roselliniamaritima*MFLUCC 24-0564 (ex-isotype): ITS = PQ778939.

##### Notes.

The construction of phylogenies from combined ITS, LSU, *tub*2 and *rpb*2 sequence data showed that *Roselliniamaritima* (MFLUCC 24-0562) formed a subclade to a group of *Rosellinia* species: *R.sanctae-cruciana* (HAST 90072903), *R.nectrioides* (CBS 449.89), *R.abscondita* (CBS 447.89), *R.marcucciana* (MUCL 51704), *R.mammiformis* (CBS 445.89) and *R.britannica* (MFLU 17-0987, HKAS 102349) in both maximum likelihood and Bayesian analyses with 85% ML/0.95 BPP statistical support showing the same topology. In a BLAST search in GenBank, the closest match of the ITS sequence of *R.maritima* (MFLUCC 24-0562) was 95% similar across 66% of the query sequence to *Rosellinia* sp. isolate (OTU1178), which was obtained using Illumina sequencing, and its morphology is not available (Gao et al. 2020). In a BLAST search in GenBank, the closest match of the LSU sequence of *R.maritima* (MFLUCC 24-0562) was 98.48% similar across 94% of the query sequence to *R.sanctae-cruciana* strain BB. *Roselliniamaritima* (MFLU 24-0452) is not comparable with *Roselliniasanctae-cruciana* as the morphology of its asexual morph is not available. Asexual morphs of *Rosellinia* have been recorded to be geniculosporium-like, dematophora-like, or nodulisporium-like (Petrini 2013), which is different from *R.maritima* (MFLU 24-0452) in having distinguished conidiophores, monoblastic geniculate conidiogenous cells, and ellipsoidal conidia. *Roselliniamaritima* (MFLUCC 24-0562) has morphological similarities with *R.truncatispora* in culture, producing pale brown, thick-walled, septate hyphal sterile elements. However, it differs by having sterile globules and aleurioconidia in culture (Fournier et al. 2017). Therefore, we introduced our collection of *R.maritima* (MFLU 24-0452) as a novel species based on its distinct morphology and phylogenetic evidence.

#### ﻿ Musicillium theobromae

Taxon classificationFungiGlomerellalesPlectosphaerellaceae

(Turconi) Zare & W. Gams

353ED20F-CA12-5B68-9AD7-3E149454AC64

Index Fungorum: IF510697

##### Description.

**Figure 7.**
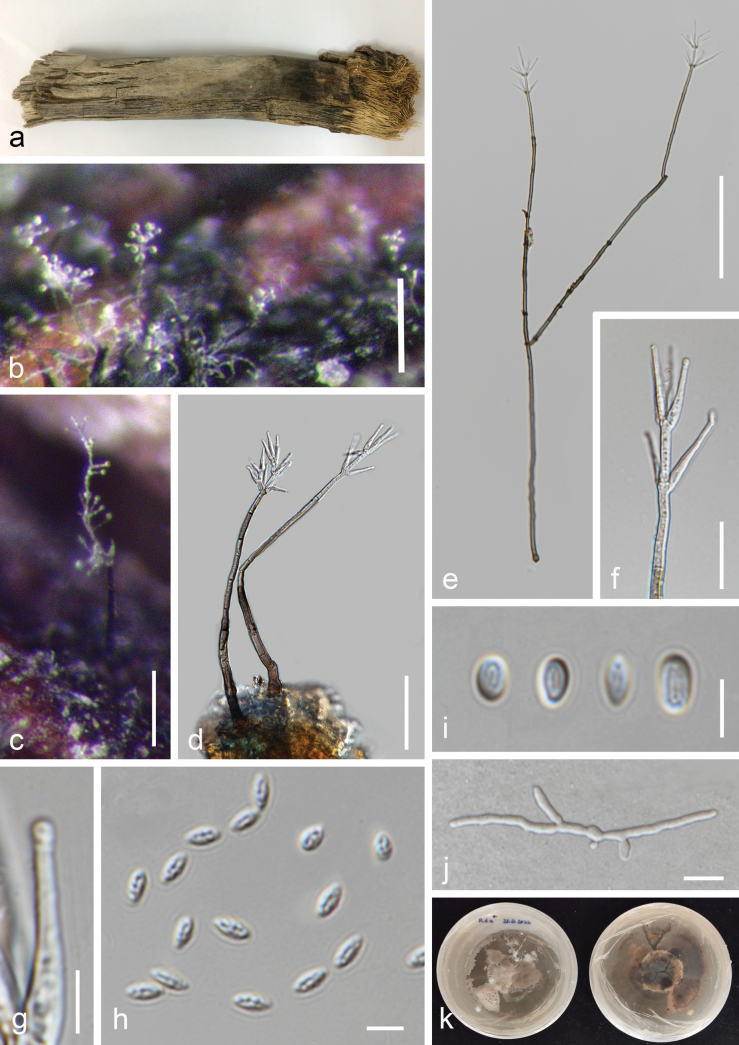
*Musicilliumtheobromae* (MFLU 24-0454, a new host record) **a** appearance of colony on the host **b, c** a close appearance of colony on the host **d, e** conidiophores **f** conidiogenous cells and developing conidia **g** close up of the phialide with an inconspicuous collarette **h, i** conidia **j** germinated conidium **k** colony on PDA after two months. Scale bars: 100 µm (**b,e**); 200 µm (**c**); 50 µm (**d**); 20 µm (**f**); 5 µm (**g–i**); 10 µm (**j**).

**Figure 8.**
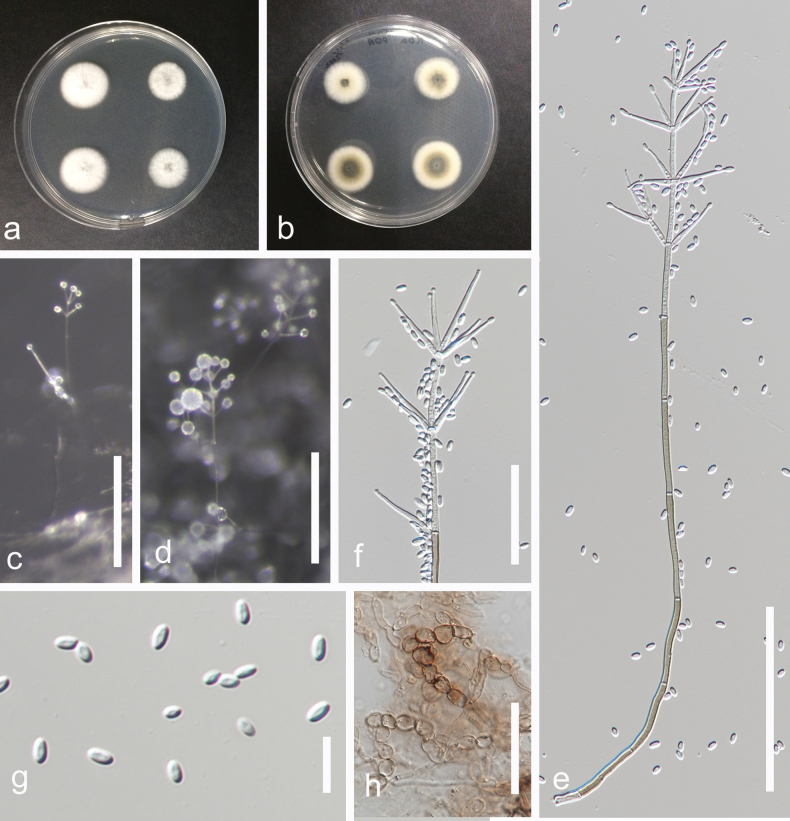
*Musicilliumtheobromae* (MFLUCC 24-0563, a new host record) **a** colony on PDA after two weeks (above) **b** colony on PDA after two weeks (below) **c, d** appearance of conidiophores and conidia on the culture **e** conidiophore and conidia **f** conidiogenous cells bearing conidia **g** conidia **h** moniliform hyphae. Scale bars: 200 µm (**c**); 100 µm (**d, e**); 50 µm (**f,h**); 10 µm (**g**).

***Saprobic*** on decaying branches of *Areca* sp. **Sexual morph**: Undetermined. **Asexual morph**: ***Hyphomycetous*** (*in vivo*, Fig. 7). ***Colonies*** on natural substrates hairy, black, with glistering white conidial masses. ***Conidiophores*** 108–503 × 3–7 µm (x– = 252 × 4.5 μm, n = 15), solitary or in groups, macronematous, mononematous, mostly straight or slightly flexuous or bent, wider at the base, unbranched or branched, septate, thick-walled, smooth-walled or slightly asperulate, brown, pale brown to subhyaline or hyaline near the apex, bearing whorls of 3–6(–7) phialides. ***Conidiogenous cells*** 13.5–27 × 2–3 µm (x– = 19 × 2.5 μm, n = 20), monophialidic, subhyaline to hyaline, cylindrical, wider at the base and tapering towards the tips, sometimes with an inconspicuous collarette, asperulate. ***Conidia*** 3.1−4.8 × 1.4−2.1 µm (x– = 3.9 × 1.8 μm, n = 40), forming in slimy heads, hyaline, cylindrical, obovoid or ellipsoidal, thick-walled, guttulate, verrucose to granular. ***Hyphomycetous*** (*in vitro*, Fig. 8). ***Colonies*** on PDA. ***Vegetative hyphae*** subhyaline, pale brown to brown, sometimes aggregating into microsclerotium-like structures, moniliform hyphae are present. ***Conidiophores*** 178–375 × 2.5–4 µm (x– = 255 × 3 μm, n = 15), arising from vegetative hyphae, mostly straight or slightly flexuous or bent, slightly tapering toward the upper part, unbranched or branched, thick-walled, septate, smooth-walled, sometimes slightly asperulate, brown to pale brown, subhyaline or hyaline at upper part, bearing phialides. ***Conidiogenous cells*** 21–35(–43) × 2–4 µm (x– = 29 × 2 μm, n = 20), phialidic, in whorls of 1–6 phialides, smooth-walled, sometimes slightly asperulate, subhyaline to hyaline, cylindrical, wider at the base and tapering towards the tips. ***Conidia*** 3−6(−7.5) × 2−4 µm (x– = 4.8 × 2.8 μm, n = 40), forming in slimy heads, hyaline, obovoid or ellipsoidal to cylindrical, thick-walled, guttulate, smooth-walled.

##### Culture characteristics.

Conidia germinating on PDA within 12 h. Colonies on PDA reaching 2 cm diam. after two weeks and 4 cm diam. after two months at 25 ± 2 °C, finely floccose, round, entire margin to finely rhizoid, white and gray brown at the center, becoming raised and brown with age. Reverse white with gray brown at the center, becoming darker with age.

##### Material examined.

Thailand, Prachuap Khiri Khan Province, Pranburi, on decaying *Areca* wood submerged in seawater and trapped between rocks, 25 October 2022, K.D. Hyde, R6a (MFLU 24-0454), living culture (MFLUCC 24-0563).

##### GenBank numbers.

*Musicilliumtheobromae*MFLUCC 24-0563: ITS = PQ778936, LSU = PQ778931, *rpb*2 = PQ885480, *tef*1-α = PQ885481.

##### Notes.

The reconstruction of phylogenies from LSU-ITS-*tef*1-α-*rpb*2 sequences showed that *Musicilliumtheobromae* (MFLUCC 24-0563) formed a sister clade with *M.theobromae* (CBS 968.72) in both maximum likelihood and Bayesian analyses with 100% ML/1.00 BPP statistical support (Fig. 3). In a BLAST search in GenBank, the closest match of the ITS sequence of *M.theobromae* (MFLUCC 24-0563) was 99.33% similar across 99% of the query sequence to *M.theobromae* (NZ62). In a BLAST search in GenBank, the closest match of the LSU sequence of *M.theobromae* (MFLUCC 24-0563) was 99.89% similar across 98% of the query sequence to *M.theobromae* (CBS 243.74). *Musicilliumtheobromae* (MFLUCC 24-0563) has similar morphology to the neotype species of *M.theobromae* (CBS 968.72), in having slightly asperulate brown septate conidiophores bearing whorls of up to six phialides, almost the same length (21–35(–43) µm vs. 17−35 µm) of phialide, which are tapering towards the tips, hyaline smooth-walled conidia forming in a slimy head (Zare et al. 2007). Therefore, *M.theobromae* (MFLUCC 24-0563) is introduced as a new host record on *Areca* based on morphology and phylogenetic evidence.

#### ﻿ Sarocladium gamsii

Taxon classificationFungiAscomycotaSordariomycetes

A. Giraldo, Gené & Guarro

E1050809-0D5B-5A61-A638-83720D8A7370

Index Fungorum: IF807944

##### Description.

**Figure 9.**
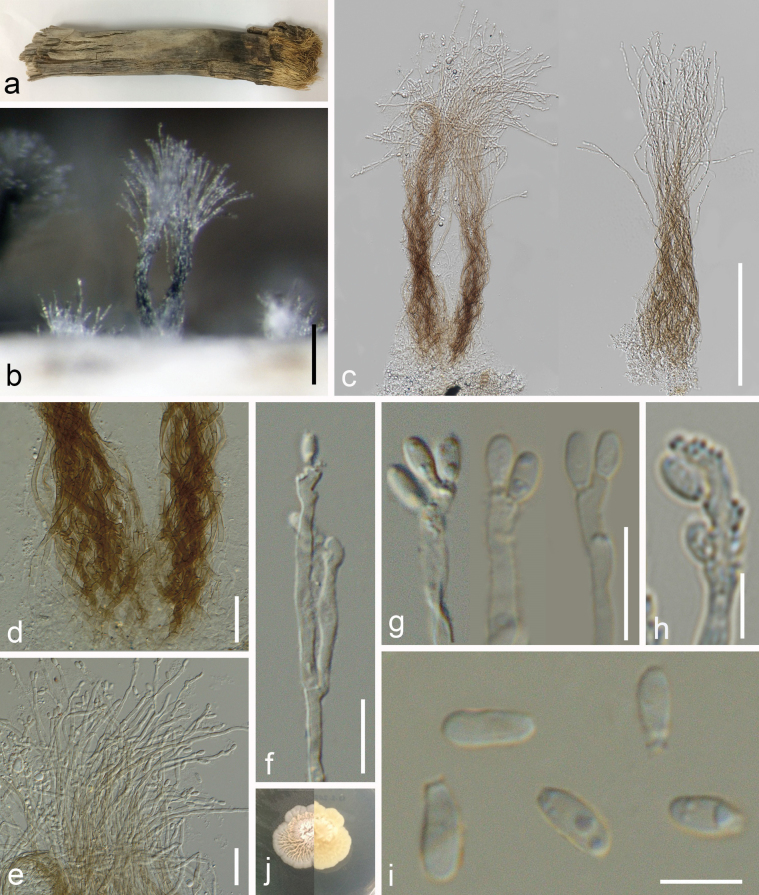
*Sarocladiumgamsii* (MFLU 24-0451, a new host record) **a** colonies on substrate **b** close-up of colonies on substrate **c** synnemata **d** base of synnemata **e** apex of synemata **f**, **g** conidiogenous cells and conidia **h** conidiogenous cell and conidia, indicating denticles at the attachment site of conidia **i** conidia **j** colony on PDA after one month (above and below). Scale bars: 100 µm (**b, c**); 20 µm (**d, e**); 10 µm (**f, g**); 5 µm (**h, i**).

**Figure 10.**
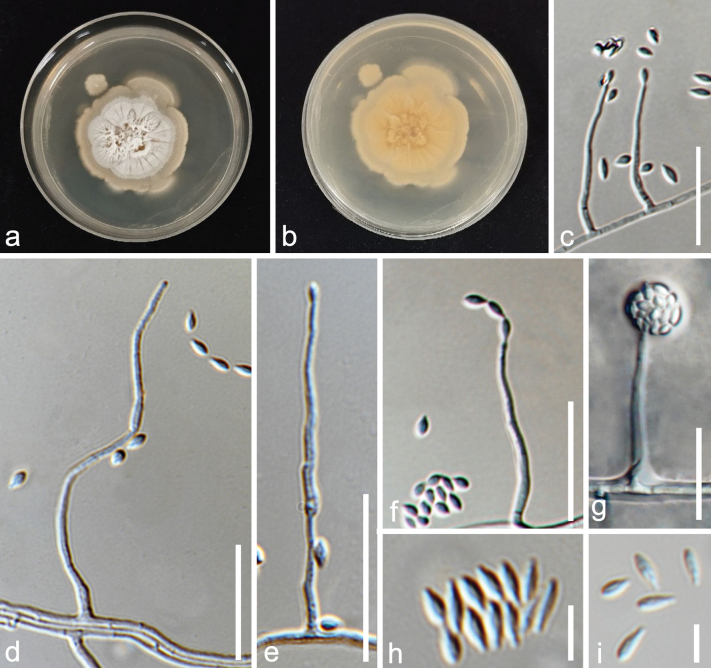
*Sarocladiumgamsii* (MFLUCC 24-0561, a new host record) **a, b** colonies on PDA after two months **c–e** conidiophores and conidia **f** conidia arranged in chains **g** conidia arranged in slimy heads **h, i** conidia. Scale bars: 20 µm (**c–e**); 5 µm (**h, i**).

***Saprobic*** on decaying branches of *Areca* sp. **Sexual morph**: Undetermined. **Asexual morph**: ***Hyphomycetous*** (*in vivo*, Fig. 9). ***Colonies*** on natural substrates hairy, effuse, scattered, grayish, shiny, with black conidiophores. ***Synemata***, tree-like, 240–280 µm height (x– = 328 μm, n = 10), wider at the base 25–62 µm wide (x– = 44 μm, n = 10), narrower in the middle 17–30 µm wide (x– = 24 μm, n = 10), divergent in the upper part, composed of 8–20 conidiophores at the base of each stipe. ***Conidiophores*** 193–298 × 1.5–3 µm (x– = 259 × 2.5 μm, n = 15), solitary or in groups, macronematous, synnematous, twisted in the stipe, parallel in the upper part, irregularly branched, straight or slightly flexuous, septate, smooth, brown, subhyaline to hyaline at the upper part. ***Conidiogenous cells*** 10–39 × 1.5–2.5 µm (x– = 21 × 2 μm, n = 15), polyphialidic, integrated or terminal, cylindrical, straight to slightly curved or sympodial at the upper part, acropetally proliferating, subhyaline to hyaline, finely aculeate, denticle at the attachment site of conidia, without collarette. ***Conidia*** 2−6.5 × 1.5−2.5 µm (x– = 4 × 2 μm, n = 30), unicellular, fusiform, truncate at base, rounded apex, hyaline, thick-walled, smooth-walled, guttulate, bud scars or disjunctors present at the site of attachment. ***Hyphomycetous*** (*in vitro*, Fig. 10). ***Colonies*** on PDA. ***Vegetative hyphae*** hyaline, smooth-walled, thin-walled, septate. ***Conidiophores*** 8.5–47(–76) µm (x– = 29 μm, n = 15) height, arising from vegetative hyphae or ropes of hyphae, straight, flexuous or slightly bent, slightly tapering toward the apex, unbranched to rarely branched, smooth-walled to asperulate, hyaline, aseptate or uniseptate at the base. ***Conidiogenous cells*** 5.5–45 × 1–1.75 µm (x– = 27 × 1.3 μm, n = 15), monophialidic, acicular, with apical periclinal thickening, finely asperulate, hyaline. ***Conidia*** 3−5 × 1−2 µm (x– = 4.5 × 1.5 μm, n = 30), solitary or forming in slimy heads or chains, hyaline, unicellular, fusiform, thick-walled, guttulate, finely asperulate. ***Chlamydospores*** not observed.

##### Culture characteristics.

Conidia germinating on PDA within 12 h. Colonies on PDA reaching 2.5 cm diam. after one month and 3 cm diam. after two months at 25 ± 2 °C, umbonate, radially folded, lobate to irregular, yellowish white. Reverse buff.

##### Material examined.

Thailand, Prachuap Khiri Khan Province, Pranburi, on decaying *Areca* wood submerged in seawater and trapped between rocks, 25 October 2022, K.D. Hyde, R6d (MFLU 24-0451), living culture (MFLUCC 24-0561).

##### GenBank numbers.

*Sarocladiumgamsii*MFLUCC 24-0561: ITS = PQ778937, LSU = PQ778932.

##### Notes.

The reconstruction of phylogenies from combined LSU, ITS and *act* sequences showed that *Sarocladiumgamsii* (MFLUCC 24-0561) formed a sister clade with *S.gamsii* (CBS 707.73 in both maximum likelihood and Bayesian analyses with 100% ML/1 BPP statistical support (Fig. 4). In a BLAST search in GenBank, the closest match of the ITS sequence of *S.gamsii* (MFLUCC 24-0561) was 99% similar across 95% of the query sequence to *S.gamsii* (PUPYF263_ANAMORPH). In a BLAST search in GenBank, the closest match of the LSU sequence of *Sarocladiumgamsii* (MFLUCC 24-0561) was 99% similar across 100% of the query sequence to *Monocillium* sp. (CBS 187.80). *Sarocladiumgamsii* (MFLUCC 24-0561) has similar morphology to *S.gamsii* (CBS 707.73) in having straight or slightly bent, unbranched or rarely branched conidiophores arising from vegetative hyphae or ropes of hyphae, monophialidic conidiogenous cells with apical periclinal thickening, fusiform, hyaline, aseptate conidia forming in chains or slimy heads, but it differs in having finely asperulate conidiogenous cells and conidia (Giraldo et al. 2015). *Sarocladiumgamsii* (MFLUCC 24-0561) is introduced as a new host record based on morphology and phylogenetic evidence.

## ﻿Discussion

Marine fungi are a taxonomically diverse group, playing crucial ecological roles as saprobes, endophytes, symbionts, and pathogens (Hyde and Jones 1988; Hyde and Lee 1998; Jones et al. 2019; Calabon et al. 2023). Saprobic lignicolous fungi are the most extensively studied group of marine fungi (Jones et al. 2019). Ascomycota is the dominantly documented marine fungal group, including the major classes Dothideomycetes and Sordariomycetes, which are suggested to have evolved from terrestrial ancestors (Vijaykrishna et al. 2006). In this study, we continue our previous investigations into fungal presence on decaying *Areca* branches submerged in seawater. Species identification was performed using both morphological and molecular data, as both methods are essential in fungal taxonomy (Chethana et al. 2021; Maharachchikumbura et al. 2021). This research has led to novel discoveries of dothideomycetous and sordariomycetous fungi in this habitat, which are discussed herein.

*Tetraploa* (Tetraplosphaeriaceae, Pleosporales, Dothideomycetes) was introduced by Berkeley and Broome (1850). Initially, it was known by its 3–4 columnar hyphomycetous conidia. Later, it was linked to a massarina-like sexual morph (Tanaka et al. 2009; Hyde et al. 2013). The genus comprises 38 species isolated from various plant materials (Species Fungorum, accessed in April 2025). There are records of *Tetraploa* from palm hosts, such as *Tetraploapalmae* (Zhang et al. 2024), but without any records on *Areca* (Xiong et al. 2024). *Tetraploa* is mostly found in terrestrial and freshwater habitats, with a few records from marine environments, such as *Tetraploaaristata* on salt marsh hosts (Calabon et al. 2021; Zhang et al. 2024). We introduce our collection of *Tetraploa* as a novel species, *T.maritima*, found on *Areca* in a marine environment in Thailand. Phylogenetic analyses of combined LSU, ITS, *tub*2 and SSU sequence data showed separation of our collection (MFLUCC 24-0565) from other *Tetraploa* species, forming a subclade to *T.pseudoaristata* (NFCCI 4624, NFCCI 4625, NFCCI 4626). The topology of our phylogenetic tree is generally consistent with previous studies (Zhang et al. 2024; Zhao et al. 2024), although there are some differences that may be attributed to the use of different sequence combinations and taxon sampling. The molecular result is further supported by morphological differences.

*Rosellinia* (Xylariaceae, Xylariales, Sordariomycetes) was described by De Notaris (1844) to accommodate its sexual type species, *R.aquila*. The genus comprises around 150 species (Li et al. 2024), with some reported from palms, such as *R.palmae*, *R.rhopalostylidicola*, and *R.calami*, though there are no records for *Areca* species (Xiong et al. 2024). *Rosellinia* has been reported from various habitats, including marine environments (Gessner and Goos 1973). The asexual morph of *Rosellinia* is known to be geniculosporium-, dematophora-, or nodulisporium-like with hyphomycetous synnemata or conidiophores forming around stromata on their natural substrates. These synnemata or conidiophores may be sterile or fertile, with the fertile parts developing geniculate holoblastic conidiogenous cells that produce ellipsoidal conidia with truncate bases (Tang et al. 2009; Petrini 2013; Fournier et al. 2017). Based on phylogenetic analyses (Fig. 2), our collection of *Roselliniamaritima* (MFLUCC 24-0562) forms a distinct clade related to *R.sanctae-cruciana* (HAST 90072903), but its morphology differs from other known asexual morphs within the genus. This distinction is seen in its production of brown to black sterile globules with a conidial-like structure composed of interwoven irregular hyphal-like cells, that mostly attach directly to the mycelium, without distinct conidiophores. The mycelium of *R.maritima* in cultures is similar to *R.truncatispora* in having brown, thick-walled, hyphal sterile elements; however, it differs by forming dark-pigmented globose structures and aleurioconidia (Fournier et al. 2017). Due to the lack of molecular data, *R.truncatispora* cannot be compared phylogenetically. Producing intertwined or moniliform, dark brown, thick-walled hyphal sterile elements in culture is also observed in *Dematophoraacutispora* and *D.asperata*, indicating the presence of such structures within the Xylariaceae (Fournier et al. 2017). However, since the *Rosellinia* (MFLU 24-0452) collection in this study was distinctly different from other asexual morphs in this genus, pictures of ex-type *R.maritima* (MFLUCC 24-0562) are provided showing the conidial-like structure, resembling those in the natural environment (Fig. 6h–x). Additionally, to minimize any possibility of contamination during the molecular process, the specimen was carefully re-examined and single spore isolation was repeated. An isotype (MFLU 24-0453) and ex-isotype (MFLUCC 24-0564) were provided. The results confirmed the morphology and sequences of the holotype (MFLU 24-0452) and ex-type (MFLUCC 24-0562).

*Musicillium* (Plectosphaerellaceae, Glomerellales, Sordariomycetes) was introduced by Zare et al. (2007) to accommodate a verticillium-like species, with *M.theobromae* designated as the type species. There are three other species included in this genus: *M.elettariae*, *M.pandanicola* and *M.tropicale* (Species Fungorum, accessed April 2025). *Musicilliumtheobromae* is a widespread species known as a pathogenic agent of cigar-end rot, frequently reported on banana (Zare et al. 2007; Youssef et al. 2020). Based on morphological and molecular evidence, our collection of *M.theobromae* (MFLUCC 24-0563) resembles *M.theobromae* (CBS 968.72). Although there have been other reports of this species on members of the palm family (Capdet and Romero 2010), in this study, we introduce our collection of *M.theobromae* (MFLUCC 24-0563), as a new host record on *Areca* (Xiong et al. 2024). Additionally, this is the first collection of *Musicillium* in a marine environment (www.marine.org; Jones et al. 2019; Calabon et al. 2023). We also provide an illustration and description of *M.theobromae* (MFLU 24-0454) from its natural substrate, which is similar to its morphology in culture, with some differences in the measurements of conidiophores, conidiogenous cells and conidia.

*Sarocladium* (Sarocladiaceae, Hypocreales, Sordariomycetes) is a hyphomycetous genus introduced by Gams and Hawksworth (1976). The genus includes 35 species reported as pathogens, endophytes, and saprobes from diverse environments (Species Fungorum, accessed April 2025; Gams and Hawksworth 1976; Yeh and Kirschner 2014; Phukhamsakda et al. 2020). A few species, such as *S.strictum* and *S.kiliense*, have been found in marine habitats (Jones et al. 2019). *Sarocladiumgamsii* was introduced by Giraldo et al. (2015) for saprobic isolates characterized by conidial arrangements in chains and slimy heads. Our collection of *Sarocladium* (MFLUCC 24-0561) clustered with *S.gamsii* (CBS 707.73) with strong molecular support, and its morphological similarity—forming conidia in chains and slimy heads—further confirms its identification as *S.gamsii*. *Sarocladiumgamsii* is presented here as a new host record on *Areca*, submerged in seawater. We also provide an illustration and description of *S.gamsii* (MFLU 24-0451), from its natural substrate, which differs from its morphology in culture. The synematous conidiomata observed match the synnematous morphology of *S.clematidis* (MFLU 17–1507), supporting the identification of this genus in its natural habitat (Phukhamsakda et al. 2020).

The four genera examined in this study—*Tetraploa*, *Rosellinia*, *Musicillium*, and *Sarocladium*—along with the previously examined genus *Myrmecridium* (Asghari et al. 2023), represent fungal groups with diverse ecological roles, ranging from saprobes to pathogens. Although these genera are mostly found in terrestrial or freshwater habitats, their presence on decaying *Areca* material submerged in seawater shows their adaptability to marine environments, which have high salinity. The discovery of three novel species and two new host records on *Areca* further demonstrates the diversity of marine fungi and suggests their ability to colonize palm substrates. Further research of this habitat may reveal more novel species and extend our knowledge of fungal diversity and their ecological functions.

## Supplementary Material

XML Treatment for Tetraploa maritima

XML Treatment for Rosellinia maritima

XML Treatment for Musicillium theobromae

XML Treatment for Sarocladium gamsii

## ﻿Additional information

### Author ORCIDs

Raheleh Asghari https://orcid.org/0009-0006-4897-5327

Chayanard Phukhamsakda https://orcid.org/0000-0002-1033-937X

E. B. Gareth Jones https://orcid.org/0000-0002-7286-5471

Ali Bahkali https://orcid.org/0000-0003-3936-0436

Carlo Chris S. Apurillo https://orcid.org/0000-0003-4348-0887

Omid Karimi https://orcid.org/0000-0001-9652-2222

Pattana Kakumyan https://orcid.org/0000-0001-8281-1082

Kevin D Hyde https://orcid.org/0000-0002-2191-0762

### Data availability

All of the data that support the findings of this study are available in the main text.
